# Sex Differences in Clinical Features in Gout: A Nationwide Retrospective Cohort Study

**DOI:** 10.3390/jcm13237095

**Published:** 2024-11-24

**Authors:** Hyunsue Do, Kwanyoung Choe, Min Jung Kim, Kichul Shin, Ki Won Moon

**Affiliations:** 1 Division of Rheumatology, Department of Internal Medicine, Kangwon National University School of Medicine, 156 Baengnyeong-ro, Chuncheon-si 24289, Republic of Korea; 2 Yeongjusi Health Center, 19 Sicheong-ro 1beon-gil, Yeongju-si 36132, Republic of Korea; 3 Division of Rheumatology, Department of Internal Medicine, Seoul Metropolitan Government-Seoul National University Boramae Medical Center, 20 Boramae-ro 5-gil, Dongjak-gu, Seoul 07061, Republic of Korea

**Keywords:** gout, sex difference, mortality, cause of death

## Abstract

**Background:** Patients with gout have several coexisting conditions that impact mortality. We analyzed the differences in clinical manifestations among Korean patients with gout and compared the causes of death based on sex using data from the Korean National Health Insurance Service-National Sample Cohort database. **Methods:** We included adults with gout receiving urate-lowering therapy (ULT) from 2002 until 2019. The clinical features and causes of death were compared between male and female patients. Multivariate Cox regression was performed to identify the risk factors contributing to all-cause mortality. **Results:** The results showed that female patients were older at the start of ULT and had more comorbidities. The most common cause of death among all patients with gout was chronic kidney disease (CKD). When observed separately by sex, lung cancer is the leading cause in males, versus CKD in females. Multivariate Cox analysis showed that old age at ULT start, low body mass index (BMI), current smoking, diabetes, CKD, cerebrovascular disease, malignancy, and low hemoglobin were significant risk factors for all-cause mortality in males; however, old age at ULT start, low BMI, CKD, malignancy, and low hemoglobin were significant risk factors in females. **Conclusions:** The clinical features and cause of death were different between male and female patients with gout, suggesting that treatment strategies for gout should be established differently depending on sex.

## 1. Introduction

Gout is an inflammatory arthritis that occurs when uric acid crystals crystallize in the joints, and it is frequently linked to elevated levels of uric acid in the body [1,2,3]. The management of pain and inflammation in acute and chronic gout typically includes nonsteroidal anti-inflammatory drugs (NSAIDs), colchicine, and corticosteroids. For patients who are unresponsive to or have contraindications for these treatments, interleukin-1 (IL-1) inhibitors may be considered as an alternative option [4]. The incidence rate of gout has increased in recent decades [5]. Gout is well known to have a significantly higher prevalence rate in men than in women [6], and investigation of female patients with gout has not been actively conducted. According to previous research findings, female patients with gout develop the disease at a later age compared to males and display a higher occurrence of comorbidities. Hypertension, diabetes mellitus (DM), and heart failure were found to have a higher prevalence among female patients with gout compared to males. Another study indicated that chronic kidney disease (CKD) also had a higher occurrence rate among women [7,8,9]. Additionally, previous studies revealed that patients with gout have an elevated overall mortality rate compared to controls, especially in men [9,10]. Male patients with gout had a susceptibility to cardiovascular deaths, particularly those stemming from coronary heart disease [10,11,12,13,14]. There have been a few studies regarding the mortality of female patients with gout; however, insufficient data are available due to the limited number of patients. In this study, we investigated the sex-based differences in clinical manifestations in patients with gout using a nationwide retrospective cohort database. Furthermore, we compared the common causes of death between male and female patients and identified significant risk factors for all-cause mortality in male and female gout patients, respectively.

## 2. Materials and Methods

### 2.1. Data Sources

This study was based on patient data collected from the Korean National Health Insurance Service-National Sample Cohort (NHIS-NSC) database. The NHIS-NSC included 2.2% of the total Korean population in 2002, following them up for 20 years until 2021. The NHIS-NSC database consists of demographic information, diagnosis code, operation, procedures, prescribed medication, and medical costs. It also contains the results of national health examinations for approximately 70% of the total participants, including lifestyle habits, physical examinations, and regular blood tests. Mortality data from the Korean National Statistical Office (the date and cause of death according to the International Classification of Disease 10th edition [ICD-10] codes) were linked to the NHIS-NSC database [15]. The present study protocol was reviewed and approved by the Institutional Review Board of Kangwon National University Hospital (approval no.: KNUH_2023-11-004, ethical approval date: 22 November 2023). This study was conducted in accordance with good clinical practice and the principles of the Declaration of Helsinki.

### 2.2. Study Population

The study enrolled individuals aged ≥ 20 years who were diagnosed with gout and who had received urate-lowering therapy (ULT: allopurinol, febuxostat, or benzbromarone) at least once. Diagnosis of gout was made when the ICD-10 code (M10) was registered. We enrolled gout patients from 1 January 2002 until 31 December 2019. The date of first ULT initiated was considered the index date, and the wash-out period was 1 year after the index date. The observation period was from 1 January 2003 to 13 December 2021. The end of follow-up was defined as when the subject died or was followed up until the end of the observation period. National health examination results closest to the index date were used for analysis, including body mass index (BMI), smoking, alcohol consumption, systolic and diastolic blood pressure (BP), hemoglobin, creatinine, fating blood sugar, total cholesterol, high-density lipoprotein (HDL), low-density lipoprotein (LDL), and triglyceride.

### 2.3. Definition of Covariates

Comorbidities were defined based on the ICD-10 code if it was recorded two or more times before the index date. They included hypertension (ICD-10 codes I10 to I15), diabetes mellitus (DM, ICD-10 codes E10 to E14), chronic kidney disease (CKD, ICD-10 codes N17 to N19), cerebrovascular disease (CVD, ICD-10 codes I60 to I69), ischemic heart disease (IHD, ICD-10 codes I20 to I25), and malignancy (ICD-10 codes C00 to C97). The date and cause of death were obtained from the NHIS-NSC dataset. The causes of death were classified according to the Korean standard classification of diseases and mortality by the Korean National Statistical Office.

### 2.4. Statistical Analysis

To compare characteristics, we calculated the frequencies and percentages for each categorical variable and the means and standard deviations (SDs) for continuous variables. We used chi-square tests and independent t-tests for categorical and continuous variables, respectively. We used the Kolmogorov–Smirnov test to check for the normal distribution of continuous variables. Univariate Cox regression analysis was performed to find significant risk factors for all-cause mortality in both male and female subjects. Multivariate Cox regression analysis was used to calculate the hazard ratio (HR) for all-cause mortality after adjusting age at ULT start, BMI, alcohol, smoking, comorbidities (hypertension, DM, hyperlipidemia, CKD, CVD, IHD, malignancy), systolic and diastolic BP, hemoglobin, creatinine, total cholesterol, high-density lipoprotein (HDL), low-density lipoprotein (LDL), and triglyceride. Cases with missing values were excluded from the Cox regression analysis to reduce bias, and the Schoenfeld test was conducted for each variable to assess the proportional hazards assumption. All statistical analyses were conducted using SAS software version 9.4 (SAS Institute, Cary, NC, USA). The level of statistical significance was set at *p* < 0.05.

## 3. Results

### 3.1. Clinical Characteristics of Patients

The total number of patients with gout who received ULT was 18,954, including 15,838 males and 3116 females. Table 1 describes the baseline characteristics of the study participants. The mean (±SD) age at ULT initiation was 51.2 (±14.6) years in males and 60.4 (±14.9) years in females, and was significantly higher in women (*p* < 0.0001). The mean (±SD) body mass index (BMI) was 25.4 (±3.3) kg/m^2^ for males and 24.8 (±3.8) kg/m^2^ for females, being significantly higher in men (*p* < 0.0001). The proportion of current smokers was higher in men than in women (37.9% vs. 4.9%, *p* < 0.0001); similarly, the proportion of moderate drinkers who drink three or more times a week was higher in men (28.4% vs. 4.5%, *p* < 0.0001).

Comorbidities were more prevalent in women than men, including hypertension (78.1% vs. 64.3%, *p* < 0.0001), DM (72.0% vs. 55.3%, *p* < 0.0001), CKD (32.8% vs. 18.9%, *p* < 0.0001), malignancy (27.4% vs. 20.3%, *p* < 0.0001), cerebrovascular disease (CVD) (41.6% vs. 24.4%, *p* < 0.0001), and ischemic heart disease (IHD) (54.1% vs. 37.3%, *p* < 0.0001).

National health examination data, including blood tests, were obtained from 13,966 (73.7%) participants. Systolic and diastolic BP were significantly higher in males than in females (128.9 ± 15.3 vs. 126.5 ± 16.8 mmHg, *p* < 0.0001 and 80.2 ± 10.4 vs. 77.3 ± 10.6 mmHg, *p* < 0.0001, respectively). The mean (±SD) creatinine level was 1.4 (±1.8) mg/dL in men and 1.2 (±1.8) mg/dL in women (*p* = 0.0031). The mean (±SD) level of triglyceride was significantly higher in men than in women (192.8 ± 159.6 vs. 150.3 ± 131.8 mg/dL, *p* < 0.0001). In contrast, the mean (±SD) level of HDL was significantly higher in women than in men (56.1 ± 30.4 vs. 51.0 ± 26.6 mg/dL, *p* < 0.0001), and that of LDL was also higher in women (118.2 ± 51.4 vs. 114.2 ± 159.4 mg/dL, *p* = 0.031). During the observation period, a total of 3703 deaths occurred. The median follow-up period of the total population was 10.3 ± 4.7 (male 10.4 ± 4.6, female 9.2 ± 4.9). The number of deaths was 2803 (17.7%) for men and 900 (28.9%) for women. The mean age at death was significantly higher in women than in men (75.4 ± 12.6 vs. 72.1 ± 12.3 years, *p* < 0.0001).

### 3.2. Common Causes of Death According to Sex

Table 2 shows the common causes of death for all patients and the differences between sexes. The overall leading cause of death was CKD, accounting for 252 (7.8%) deaths. Others were lung cancer (6.5%), DM (4.9%), myocardial infarction (4.6%), pneumonia (3.8%), malignant neoplasm of the liver and intrahepatic bile duct (3.0%), heart failure (2.6%), cerebral infarction (2.6%), malignant neoplasm of the stomach (2.4%), senility (2.3%), and other causes (59.5%). The common causes of death differed between males and females. The common causes of death in males included lung cancer (7.6%), CKD (5.2%), myocardial infarction (4.9%), pneumonia (4.4%), malignant neoplasm of the liver and intrahepatic bile duct (3.5%), malignant neoplasm of the stomach (3.3%), DM (3.3%), cerebral infarction (3.1%), other and unspecified effects of external causes (2.9%), other ill-defined and unspecified causes of mortality (2.6%), and other causes (59.4%). In contrast, the common causes of death in females were CKD (10.39%), DM (7.1%), heart failure (5.6%), senility (4.2%), lung cancer (3.9%), myocardial infarction (3.8%), pneumonia (3.6%), acute myeloid leukemia with multi-lineage dysplasia (2.3%), unspecified DM with coma (2.2%), chronic IHD (2.2%), and other causes (54.7%).

### 3.3. Cox Regression for Overall Mortality in Male and Female Gout Patients

Table 3 and Table 4 present the results of the Cox regression analysis for overall mortality in male and female gout patients, respectively. We analyzed the data after excluding cases with missing values to reduce bias. The number of male subjects was 11,822, decreased from 15,838, and that of female subjects was 2065, decreased from 3116 after deleting missing values. The Schoenfeld test for assessing Cox proportional hazards assumption for each variable was displayed in Supplementary Table S1. Multivariate analysis of male gout patients showed that age at ULT initiation (HR 1.091, *p* < 0.0001), BMI (HR 0.944, *p* < 0.0001), current smoking (HR 1.261, *p* < 0.0001), DM (HR 1.353, *p* < 0.0001), CKD (HR 1.559, *p* < 0.0001), CVD (HR 1.309, *p* < 0.0001), malignancy (HR 1.895, *p* < 0.0001), and hemoglobin (HR 0.852, *p* < 0.0001) were significant factors for all-cause mortality. However, multivariate analysis of female gout patients displayed age at ULT start (HR 1.085, *p* < 0.0001), BMI (HR 0.960, *p* = 0.0082), CKD (HR 2.310, *p* < 0.0001), malignancy (HR 1.845, *p* < 0.0001), and hemoglobin (HR 0.873, *p* = 0.0004) as significant factors for all-cause mortality (Table 4).

## 4. Discussion

This study aimed to compare clinical manifestations between male and female patients with gout in Korea and analyze the risk factors for all-cause mortality. Investigating the clinical features and cause of death in female patients with gout using a national cohort database was valuable, as data about female patients have been lacking. In previous studies, female patients with gout developed a later disease onset than males [7,16] and were associated with higher BMI [17]. Heart failure, CKD, and DM were more prevalent in female patients with gout, whereas male patients with gout were linked to obstructive respiratory diseases, coronary artery disease, and peripheral vascular disease [18]. Compared with males, females with gout exhibited a higher likelihood of obesity, elevated serum urate levels (0.53 vs. 0.49 mmol/L), a greater frequency of diuretics usage (60% vs. 30%), and lower alcohol consumption (47% vs. 72%) [19,20]. Our study showed that the rate of current smokers and moderate drinkers was higher among males, and female patients had a higher prevalence of comorbidities, including hypertension, DM, CKD, CVD, and IHD. The rate of malignancy was also higher among female patients, which has not been previously reported.

Several previous studies have reported the cause of death in patients with gout. A Swedish study reported that the causes of death in patients with gout were renal disease (HR: 1.78 [95% confidence interval{CI}: 1.34–2.35]), diseases of the digestive system (HR: 1.56 [95%CI: 1.34–1.83]), cardiovascular disease (HR: 1.27 [95% CI: 1.22–1.33]), infectious diseases (HR: 1.20 [95% CI: 1.06–1.35]), and dementia (HR: 0.83 [95% CI: 0.72–0.97]) [21]. An epidemiologic study in Taiwan showed that male and female patients with gout had higher mortality than controls, and that the main causes of death were kidney, endocrine, metabolic, and cardiovascular diseases [9]. Similarly, a Dutch study reported that patients with gout had a higher mortality rate attributed to cardiovascular diseases, infectious diseases, and cancer [22]. The association between gout and cardiovascular mortality has been well established; however, death from non-cardiovascular diseases is relatively unclear. Our study showed that the most common cause of death was CKD rather than acute illnesses. Furthermore, we compared the difference in mortality rate and causes of death between males and females. The mortality rate in patients with gout in Korea was higher in females, which might be caused by the age of ULT start, which was about 10 years older than male. The main cause of death differed between males and females. In males, the primary cause of death was lung cancer, followed by CKD, myocardial infarction, and pneumonia. Contrastingly, the leading cause of death in females was CKD, followed by DM, heart failure, and senility.

In our study, multivariate Cox regression analysis showed that significant risk factors for all-cause mortality were old age at ULT start, low BMI, current smoking, DM, CKD, CVD, malignancy, and low hemoglobin in male gout patients. However, significant risk factors in females were old age at ULT start, low BMI, CKD, malignancy, and low hemoglobin. These discrepancies might be due to sex itself or the different numbers of male and female subjects, as there were six times more males than women. Our study showed that BMI has a negative impact on survival in both male and female gout patients. There is a well-known that a J-shape relationship between BMI and all-cause mortality, and both underweight and obesity have a harmful effect on survival. But low BMI seems to increase mortality in patients with hyperuricemia. A Taiwan cohort study reported that the mortality rate was highest when BMI was less than 21 kg/m^2^ among hyperuricemic patients with uric acid levels of 6.6 mg/dL or higher [23]. Another study in patients with congestive heart failure also displayed that hyperuricemia and low BMI less than 22 kg/m^2^ increased mortality [24]. Our study also reveals that hemoglobin level was negatively correlated with all-cause mortality in both male and female gout patients. Anemia was reported to be a risk factor for predicting cardiovascular and all-cause mortality in a meta-analysis analyzing studies of the general population [25]. A cohort study of patients with CKD showed that anemia and hyperuricemia had a synergistic effect on all-cause mortality [26].

Until now, gout research has predominantly focused on males due to a higher prevalence of gout in males, resulting in limited studies about females. As shown by our study, the clinical manifestations and main causes of death differ based on sex. These findings suggest that gout management strategies should be implemented differently depending on sex. Males with gout had higher BMI, BP, creatinine, and triglyceride levels, and they comprised more current smokers and moderate drinkers. Their main causes of death were lung cancer, CKD, myocardial infarction, pneumonia, and malignancy of the liver. Therefore, regarding implications for male patients with gout, physicians should focus on lifestyle modification, including reducing body weight, refraining from heavy smoking, curbing excessive alcohol consumption, and providing treatment to prevent cardiovascular disease for male patients with gout. However, female patients had older age, more comorbidities, higher cholesterol profile, and their main causes of death were CKD, DM, heart failure, senility, and lung cancer. Thus, regarding implications for female patients with gout, treatment for lowering cholesterol profile, preventing CKD, and managing combined diseases should be emphasized in female patients with gout.

### Limitations

Although this study provides various insights, it has limitations that should be acknowledged. First, there might be racial differences in the main causes of death in patients with gout. Since the causes of death in the general population are different based on each race and country, the cause of death in patients with gout in other regions may vary compared to the East Asian ethnic group of the South Korean population. Second, cause of death may be inaccurate. We used mortality data from the NHIS-NSC database collected from the Korean National Statistical Office. Mortality data were collected from medical records from each hospital, a process that may introduce inaccuracies depending on the recordings of individual information. Third, our results may not fully represent the entire population, as this database was derived from sample cohort data rather than the entire national population. However, it is important to highlight that the sample cohort data in this study was well-designed to reflect the characteristics of the general population. This careful design increases the reliability of our study’s result; however, it is still crucial to interpret it with caution when applying it to the health of the general population. Lastly, the data used in this study may have methodological limitations for direct comparison between males and females due to the sample size disparity. Gout is a male-dominated disease, and the study of female gout patients has not been actively conducted until now. We believe our study is valuable despite these limitations in that we investigated sex differences in gout patients.

## 5. Conclusions

Clinical features and cause of death were different between male and female patients with gout. Female patients with gout were older than males and had more comorbidities, and their main causes of death were different. Lung cancer is the leading cause of death in males; on the other hand, the leading cause of death in females was CKD. These findings imply that there is a difference in clinical manifestations between males and females, and treatment strategies for gout should be tailored according to sex.

## Figures and Tables

**Table 1:** Clinical characteristics of the study population.

Characteristics	Male (n = 15,838)	Female (n = 3116)	Total Missing Value (%)	*p* Value
Age at ULT initiation	51.2 ± 14.6	60.4 ± 14.9	0.0	<0.0001 ***
BMI	25.4 ± 3.3	24.8 ± 3.8	26.3	<0.0001 ***
Smoking			26.5	<0.0001 ***
None or past smoker	7371 (62.1)	1971 (95.1)		
Current smoker	4492 (37.9)	102 (4.9)		
Alcohol consumption			26.7	<0.0001 ***
None or occasional drinkers	8476 (71.6)	1974 (95.5)		
Moderate drinker (≥3/week)	3358 (28.4)	93 (4.5)		
Comorbidities (%)			0.0	
Hypertension	10,186 (64.3)	2434 (78.1)		<0.0001 ***
Diabetes mellitus	8751 (55.3)	2243 (72.0)		<0.0001 ***
Chronic kidney disease	2998 (18.9)	1023 (32.8)		<0.0001 ***
Cerebrovascular disease	3865 (24.4)	1297 (41.6)		<0.0001 ***
Ischemic heart disease	5910 (37.3)	1686 (54.1)		<0.0001 ***
Malignancy	3218 (20.3)	854 (27.4)		<0.0001 ***
Systolic BP	128.9 ± 15.3	126.5 ± 16.8	26.3	<0.0001 ***
Diastolic BP	80.2 ± 10.4	77.3 ± 10.6	26.3	<0.0001 ***
Hemoglobin	14.7 ± 1.5	12.6 ± 1.4	26.3	<0.0001 ***
Creatinine	1.4 ± 1.8	1.2 ± 1.8	26.3	0.0031 **
Total cholesterol	197.0 ± 40.7	200.8 ± 42.3	26.3	0.0002 **
HDL	51.0 ± 26.6	56.1 ± 30.4	26.3	<0.0001 ***
LDL	114.2 ± 159.4	118.2 ± 51.4	27.4	0.0311 *
Triglyceride	192.8 ± 159.6	150.3 ± 131.8	26.3	<0.0001 ***
Death	2803 (17.7)	900 (28.9)	0.0	<0.0001 ***
Age at death	72.1 ± 12.3	75.4 ± 12.6	0.0	<0.0001 ***

Values are means ± standard deviations or numbers and frequencies (%). ULT, urate-lowering therapy, BMI, body mass index, BP, Blood pressure, HDL, high-density lipoprotein, LDL, low-density lipoprotein; *, *p* < 0.05; **, *p* < 0.01; ***, *p* < 0.001.

**Table 2:** Common causes of death in all gout patients and the differences between sex.

	Total	N (%)	Male	N (%)	Female	N (%)
1	Chronic kidney disease	252 (7.8)	Lung cancer	137 (7.6)	Chronic kidney disease	80 (10.4)
2	Lung cancer	210 (6.5)	Chronic kidney disease	93 (5.2)	Diabetes mellitus	55 (7.1)
3	Diabetes mellitus	159 (4.9)	Myocardial infarction	87 (4.9)	Heart failure	43 (5.6)
4	Myocardial infarction	147 (4.6)	Pneumonia	79 (4.4)	Senility	32 (4.2)
5	Pneumonia	123 (3.8)	Malignant neoplasm of the liver and intrahepatic bile duct	62 (3.5)	Lung cancer	30 (3.9)
6	Malignant neoplasm of the liver and intrahepatic bile duct	97 (3.0)	Malignant neoplasm of stomach	59 (3.3)	Myocardial infarction	29 (3.8)
7	Heart failure	82 (2.6)	Diabetes mellitus	59 (3.3)	Pneumonia	28 (3.6)
8	Cerebral infarction	82 (2.6)	Cerebral infarction	55 (3.1)	Acute myeloid leukemia with multi-lineage dysplasia	18 (2.3)
9	Malignant neoplasm of stomach	77 (2.4)	Other and unspecified effects of external causes	52 (2.9)	Unspecified diabetes mellitus, with coma	17 (2.2)
10	Senility	75 (2.3)	Other ill-defined and unspecified causes of mortality	46 (2.6)	Chronic ischemic heart disease	17 (2.2)
	Other causes	2405 (59.5)	Other causes	2074 (59.4)	Other causes	551 (54.7)

Values are numbers and frequencies (%).

**Table 3:** Cox regression analysis of all-cause mortality in male gout patients (n = 11,822).

	Univariate		Multivariate	
	Hazard Ratio (95% CI)	*p* Value	Hazard Ratio (95% CI)	*p* Value
Age at ULT start	1.112 (1.107–1.118)	<0.0001 ***	1.091 (1.085–1.098)	<0.0001 ***
BMI	0.862 (0.848–0.877)	<0.0001 ***	0.944 (0.927–0.962)	<0.0001 ***
Alcohol				
None or occasional drinking ^a^	ref		ref	
Moderate drinking ^b^	0.742 (0.694–0.793)	<0.0001 ***	0.996 (0.934–1.063)	0.9147
Smoking				
None or past smoking	ref		ref	
current smoking	0.844 (0.794–0.897)	<0.0001 ***	1.261 (1.178–1.349)	<0.0001 ***
Comorbidities				
Hypertension	4.550 (3.843–5.388)	<0.0001 ***	1.058 (0.876–1.279)	0.5577
Diabetes mellitus	3.728 (3.256–4.269)	<0.0001 ***	1.353 (1.168–1.567)	<0.0001 ***
Chronic kidney disease	3.870 (3.490–4.292)	<0.0001 ***	1.559 (1.388–1.751)	<0.0001 ***
Cerebrovascular disease	3.697 (3.339–4.094)	<0.0001 ***	1.309 (1.172–1.461)	<0.0001 ***
Ischemic heart disease	2.968 (2.669–3.299)	<0.0001 ***	1.113 (0.990–1.251)	0.0740
Malignancy	3.732 (3.369–4.134)	<0.0001 ***	1.895 (1.705–2.108)	<0.0001 ***
Systolic BP	1.011 (1.008–1.014)	<0.0001 ***	1.003 (0.999–1.007)	0.2011
Diastolic BP	0.984 (0.979–0.989)	<0.0001 ***	1.004 (0.997–1.010)	0.2987
Laboratory results				
Hemoglobin	0.706 (0.692–0.721)	<0.0001 ***	0.852 (0.823–0.882)	<0.0001 ***
Creatinine	1.026 (1.014–1.038)	<0.0001 ***	1.003 (0.980–1.001)	0.7834
Total cholesterol	0.992 (0.991–0.993)	<0.0001 ***	1.000 (0.998–1.001)	0.5740
HDL	0.999 (0.996–1.001)	0.2544	0.9999 (0.997–1.002)	0.6149
LDL	0.996 (0.995–0.998)	<0.0001 ***	1.000 (0.999–1.001)	0.7927
Triglyceride	0.998 (0.997–0.998)	<0.0001 ***	1.000 (1.000–1.001)	0.8350

CI, confidence interval, ULT, urate-lowering therapy, BMI, body mass index, BP, blood pressure, HDL, high-density lipoprotein, LDL, low-density lipoprotein, ^a^ non-drinking and light drinking (1–2 times/week), ^b^ moderate drinking (≥3 times/week), *** *p* < 0.001.

**Table 4:** Cox regression analysis of all-cause mortality in female gout patients (n = 2065).

	Univariate		Multivariate	
	Hazard Ratio (95% CI)	*p* Value	Hazard Ratio (95% CI)	*p* Value
Age at ULT start	1.103 (1.091–1.114)	<0.0001 ***	1.085 (1.073–1.098)	<0.0001 ***
BMI	0.947 (0.912–0.979)	0.0080 **	0.960 (0.931–0.989)	0.0082 **
Alcohol				
None or occasional drinking ^a^	ref		ref	
Moderate drinking ^b^	0.495 (0.363–0.674)	<0.0001 ***	1.040 (0.764–1.417)	0.8035
Smoking				
None or past smoking	Ref		ref	
Current smoking	1.033 (0.820–1.302)	0.7833	1.145 (0.926–1.715)	0.1746
Comorbidities				
Hypertension	6.357 (4.047–9.984)	<0.0001 ***	1.275 (0.764–2.129)	0.3524
Diabetes mellitus	4.061 (2.901–5.687)	<0.0001 ***	1.343 (0.932–1.935)	0.1135
Chronic kidney disease	3.929 (3.188–4.842)	<0.0001 ***	2.310 (1.823–2.928)	<0.0001 ***
Cerebrovascular disease	2.948 (2.377–3.656)	<0.0001 ***	1.171 (0.930–1.473)	0.1790
Ischemic heart disease	2.900 (2.298–3.661)	<0.0001 ***	1.168 (0.906–1.506)	0.2316
Malignancy	2.376 (1.926–2.932)	<0.0001 ***	1.845 (1.484–2.294)	<0.0001 ***
Systolic BP	1.019 (1.013–1.024)	<0.0001 ***	1.007 (0.999–1.015)	0.0845
Diastolic BP	1.007 (0.997–1.016)	0.1897	0.997 (0.984–1.010)	0.6171
Laboratory results				
Hemoglobin	0.715 (0.671–0.763)	<0.0001 ***	0.873 (0.810–0.940)	0.0004 **
Creatinine	1.037 (1.008–1.067)	0.0120 *	0.977 (0.918–1.040)	0.4682
Total cholesterol	0.994 (0.992–0.997)	<0.0001 ***	0.998 (0.993–1.003)	0.3604
HDL	0.972 (0.964–0.980)	<0.0001 ***	0.994 (0.987–1.000)	0.0525
LDL	0.994 (0.992–0.997)	<0.0001 ***	1.000 (0.996–1.005)	0.8602
Triglyceride	1.001 (1.000–1.001)	0.0062 **	1.001 (1.000–1.002)	0.0698

CI, confidence interval, ULT, urate-lowering therapy, BMI, body mass index, BP, blood pressure, HDL, high-density lipoprotein, LDL, low-density lipoprotein, ^a^ non-drinking and light drinking (1–2 times/week), ^b^ moderate drinking (≥3 times/week), * *p* < 0.05; ** *p* < 0.01; *** *p* < 0.001.

## Data Availability

The original contributions presented in the study are included in the article/Supplementary Materials, further inquiries can be directed to the corresponding author.

## Supplementary Materials

The following supporting information can be downloaded at: https://www.mdpi.com/article/10.3390/jcm13237095/s1, Table S1. Schoenfeld test for assessing Cox proportional hazards assumption for each variable. ULT urate-lowering therapy, BMI body mass index, BP Blood pressure, HDL High-density lipoprotein, LDL Low-density lipoprotein.
