# Supplementary material for: Sex Differences in Clinical Features in Gout: A Nationwide Retrospective Cohort Study
Source: J Clin Med. 2024 Nov 24;13(23):7095. doi: 10.3390/jcm13237095 (PMC11642280; doi:10.3390/jcm13237095)
Supplement: Supplementary file 1 [file jcm-13-07095-s001.zip › jcm-3286731-supplementary.pdf]

Supplementary Table S1. Schoenfeld test for assessing Cox proportional hazards assumption for each variable. *ULT* urate-lowering therapy, *BMI* body mass index, *BP* Blood pressure, *HDL* High-density lipoprotein, *LDL* Low-density lipoprotein.

	**Male**			**Female**		
	**Chisq**	**df**	**P value**	**Chisq**	**df**	**P value**
Age at ULT start	0.702	1	0.402	0.141	1	0.180
BMI	2.970	1	0.085	1.060	1	0.304
Alcohol	0.475	1	0.491	5.550	1	0.185
Smoking	1.020	1	0.312	0.778	1	0.378
Comorbidities						
Hypertension	0.198	1	0.656	0.448	1	0.343
Diabetes mellitus	1.090	1	0.297	0.524	1	0.998
Chronic kidney disease	0.030	1	0.862	0.130	1	0.719
Cerebrovascular disease	0.349	1	0.555	0.441	1	0.357
Ischemic heart disease	0.363	1	0.547	1.830	1	0.176
Malignancy	0.222	1	0.240	1.590	1	0.650
Systolic BP	2.120	1	0.146	0.779	1	0.377
Diastolic BP	0.270	1	0.604	0.473	1	0.492
Laboratory results						
Hemoglobin	0.670	1	0.407	0.479	1	0.286
Creatinine	0.106	1	0.745	0.015	1	0.903
Total cholesterol	1.610	1	0.205	0.063	1	0.801
HDL	0.928	1	0.335	0.036	1	0.849
LDL	1.330	1	0.248	0.132	1	0.717
Triglyceride	0.009	1	0.975	0.102	1	0.749
